# Tobacco use disorder in patients with other mental disorders: a dual disorder perspective from clinical neuroscience

**DOI:** 10.3389/fpsyt.2024.1427561

**Published:** 2024-10-11

**Authors:** Nestor Szerman, Carlos Parro, Pablo Vega, Ignacio Basurte-Villamor, Miguel Ruiz-Veguilla

**Affiliations:** ^1^ World Association of Dual Disorders, WPA Section on Dual Disorders, Madrid, Spain; ^2^ Institute of Psychiatry and Mental Health, Gregorio Marañón University Hospital, Madrid, Spain; ^3^ Institute for Addictions, Madrid Salud, Madrid City Council, Madrid, Spain; ^4^ López Ibor Clinic, Madrid, Spain; ^5^ European University of Madrid, Madrid, Spain; ^6^ Virgen del Rocío Hospital, IBIS Centre for Biomedical Research in Mental Health (CIBERSAM), Seville, Spain; ^7^ University of Seville, Seville, Spain

**Keywords:** tobacco use disorder, dual disorders, biological treatments, electronic cigarettes, harm reduction

## Abstract

Tobacco smoking is the leading cause of disability and preventable deaths worldwide, but it should be differentiated from tobacco use disorder, which is, according to the Diagnostic and Statistical Manual of Mental Disorders, a *bona fide* mental disorder. The rapid delivery of nicotine to the brain activates acetylcholine receptors and stimulates the release of dopamine, both systems implicated in other mental disorders. Rates of tobacco use disorder are much higher among people suffering from other mental disorders and these patients find it more difficult to quit. Dual disorders, from a transdiagnostic perspective, identify patients with substance use disorder, in this case tobacco use disorder, and other mental disorders. A dual disorder is a complex clinical condition that is often underdiagnosed, undertreated, and difficult to manage. Appropriate and integrated tobacco use disorder treatment programs for people also suffering from other mental disorders could improve outcomes. Bio-psycho-social approaches to tobacco use disorder include specific biological treatments (e.g., bupropion, varenicline, cytisine, nicotine replacement therapy or deep trans-magnetic stimulation). However, these treatments don’t have the same outcomes in patients with dual disorders. Therefore, as in other dual disorders, harm reduction measures, such as vaping nicotine through electronic cigarettes or tobacco replacement therapies should be considered as alternative tools for dual tobacco use disorder management. These clinical considerations emerge from a narrative literature review and expert consensus and will specifically address considerations for changes in clinical practice to improve the treatment of tobacco use disorder and other mental disorders.

## Introduction

Tobacco smoking is a persistently devastating public health problem, the major preventable cause of disease and death worldwide (1). People who ever smoked have around five times greater risk of lung cancer and smoking is also a risk factor for the development of pulmonary and cardiovascular diseases, such as chronic obstructive pulmonary disease (COPD) (2). According to the World Health Organization (WHO), Europe has the highest prevalence of tobacco smoking among adults (28%) and one of the highest among adolescents (12% on average, but as high as 52% in some countries). This region also has one of the highest rates of deaths attributable to tobacco use: 16% of all deaths in adults over the age of 30.

But from a clinical perspective, it is important to differentiate tobacco use from Tobacco Use Disorder (TUD) (3). According to international classifications, TUD is recognized as a mental disorder, different from a smoking habit, characterized, according to DSM-5 TR, as a problematic pattern of tobacco use leading to clinically significant impairment or distress. Importantly, when a TUD co-occurs with other mental disorder(s) the resulting clinical condition is identified as a dual disorder (DD) (4). People suffering from other psychiatric conditions often display higher rates of tobacco smoking relative to the general population (5). Predictably, mental disorders have been linked to more severe TUD, greater withdrawal symptoms, and lower quit rates (6).

Thus, in addition to screening and treating for tobacco use, which could exacerbate an underlying mental illness (7), healthcare providers should consider screening for mental health problems as predictors of TUD: personalized interventions addressing mental health problems could prevent youth from initiating tobacco use in the first place (8). The higher TUD prevalence associated with most mental disorders is even more pronounced among people with severe mental disorders (i.e., those exhibiting a higher degree of functional impairment, namely schizophrenia, bipolar disorder, and major depressive disorder) (9).

Specific anxiety disorders and mood disorders have also been associated with higher risk of TUD (10). Studies have consistently suggested that TUD could play a contributing role in bipolar disorder where it could constitute a marker of severity (11). And an association between TUD and depression and anxiety has been reported among adolescents (12). Due to the apparent relationship between tobacco consumption and mental disorders, those individuals are more vulnerable to the development of TUD, and the negative health consequences stemming from tobacco use (6). Indeed, in the USA, people with mental illness account for almost half of the annual tobacco attributable deaths and die, on average, 25 years prematurely (13).

The high concurrency between TUD and other mental disorders cannot be explained by conceptual or measurement artifacts. Considering the growing evidence suggesting that DD are not the consequence of random or coincidental factors, it seems reasonable to explore the assertion that TUD and other mental disorders may be causally (and likely bidirectionally) linked (14, 15).

## Epidemiology

### Prevalence of TUD in patients with other mental disorders

The relationship between SUD and other mental disorders is well established. Patients with SUD represent up to 75% of patients with severe mental illness (16), while another study found an association between a lifetime diagnosis of a mental disorder and a higher prevalence of transition from substance use to SUD (17). Among the substances surveyed in this review, tobacco displayed the strongest association. Indeed, smoking rates are two- to four-fold higher in patients with mental disorders and SUD (5, 18). Studies from the US and UK estimated that people with mental disorders consume nearly 45% of all cigarettes smoked, even though their proportions in the general population are closer to 20 and 17%, respectively.

### Associations between TUD other specific psychiatric disorders

Regarding severe mental disorders, a recent meta-analysis indicated that the pooled worldwide prevalence of TUD among people with a primary diagnosis of schizophrenia, bipolar disorder, or major depressive disorder is 65%, 46.3%, and 33.4%, respectively (9). Anxiety disorders also increase the risk of TUD (19, 20). A higher prevalence of TUD has similarly been observed in patients with depression, with a plausible dose dependent relationship between the severity of addiction and depressive symptoms (21). In fact, the global increase in the prevalence of depression (a significant barrier to smoking cessation) has been proposed to be one contributor to the recent plateauing of smoking rates after decades of sharp declines. From a public health perspective, it is important to point out that tobacco control policies have not always proven to be effective for people with mental disorders (22).

Attention deficit hyperactivity disorder (ADHD) is another clinical condition tightly linked to tobacco consumption and TUD. ADHD in childhood and adolescence has been identified as a strong predictor of the use of tobacco, alcohol, and other drugs in adulthood (23). Moreover, young people with ADHD are two- to three-fold more likely to smoke tobacco regularly and develop TUD. Also, subjects with ADHD start smoking tobacco earlier and have a faster progression to a severe TUD (i.e., consuming a greater number of cigarettes per day than those without ADHD) (24). A higher prevalence of TUD is present also in people with other SUDs (25).

The epidemiological evidence of associations between TUD and other psychiatric disorders is indeed compelling (26). A better understanding of the underlying mechanisms could be the basis for improved interventions for tobacco related DDs.

## Underlying mechanisms

### Likely contributors to the association between TUD and other mental disorders

**Figure 1:**
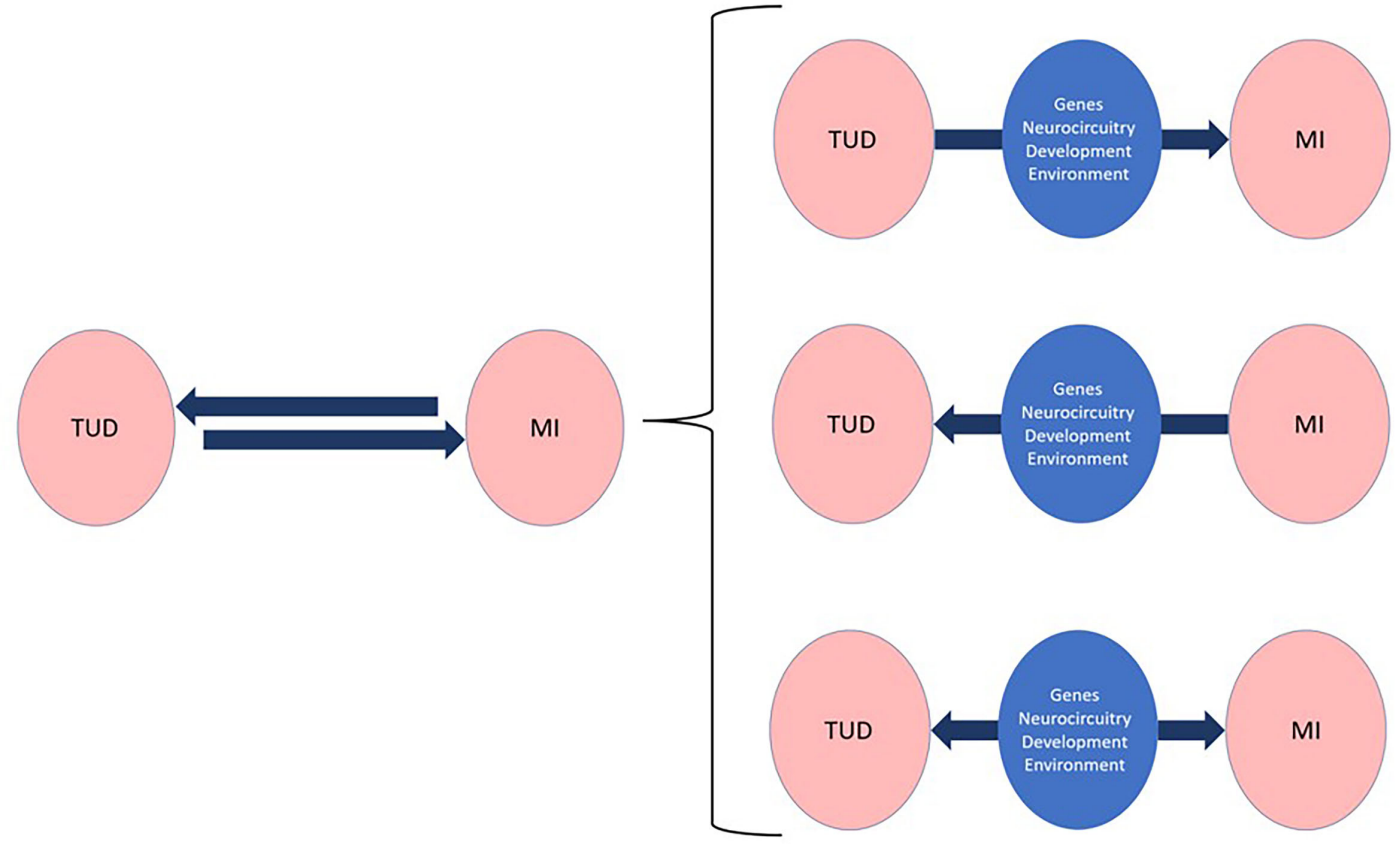
Repeated observations of strong epidemiological congruities between tobacco use disorders (TUD) and other mental illnesses (MI) suggest causal, bidirectional links that belie complex underlying mechanisms mediating most dual disorders. A better understanding of these mechanisms, which operate through different phenomenological and neurobiological levels, is bound to incentivize the development of personalized and thus more effective interventions for the prevention, diagnosis, and treatment of Dual TUD.

Several explanations have been proposed to explain the high prevalence of smoking and TUD among people with mental disorders (5). While the frequent co-occurrence of addictions in general with other psychiatric disorders suggests both conditions may be causally linked (27), the underlying mechanisms are likely complex with both mental disorders often sharing neurobiological pathways and genetic, developmental, and environmental risk factors (**Figure 1**). For that reason, DD are frequently underdiagnosed, undertreated, and can be difficult to manage (28).

Moreover, whether simultaneous or sequential, the occurrence of both disorders in one individual can affect the course and prognosis of each condition (29). This, along with the lack of studies or treatment guidelines, specifically focused on dual TUD, hinders the delivery of optimized therapeutic approaches towards dual patients. The underlying mechanisms operate at various phenomenological and neurobiological levels, as outlined below.

### Neurochemistry

In the case of co-occurring TUD with other mental disorders, it is possible that the same (i.e., the nicotinic-cholinergic system) or overlapping brain circuits are involved in both disorders. In fact, while the expression of nicotinic receptors is reduced in some mental disorders, such as depression or schizophrenia, nicotine administration can reduce aggressive behavior and agitation and may be helpful in reducing depressive symptoms (30). The specific neurocognitive deficits present in schizophrenia are a specific phenotype or endophenotype present in 80% of cases and that remains stable in all phases of the disease, constituting a vulnerability factor for the initiation and maintenance of tobacco consumption (31, 32).

These patients have a higher prevalence of smoking; they smoke more cigarettes per day, are more nicotine dependent, and smoke more intensely. The likelihood of these outcomes has been linked to a dysregulated nicotine-cholinergic system, such that patients with schizophrenia may smoke to alleviate some of the neurocognitive symptoms associated with the disorder (31, 32). Consistent with this hypothesis, a recent connectome-wide analysis has found that nicotine administration normalizes the hyperconnectivity that characterizes the default mode network of patients with TUD and schizophrenia (33). This result strongly suggests that nicotine use in schizophrenia may be a specific attempt to correct a network deficit known to interfere with cognition; it also provides direct evidence that the biological basis of nicotine dependence is different in schizophrenia and in non-schizophrenia populations (33). These findings can also help predict an individual’s response to the use of various substances and, therefore, in designing targeted prevention interventions, the expressed goal of precision psychiatry (34).

However, research shows that TUD is due to more than nicotine alone. One hypothesis is that monoamine oxidase (MAO) inhibition from non-nicotinic components in, or derived from, tobacco smoke contributes to TUD. On the other hand, interestingly, the ability of MAO inhibitors to ameliorate dopaminergic deficits underlies their clinical potential to treat depression, anxiety, and Parkinson’s disease (35).

### Self-medication hypothesis

Combined, the above and other observations are consistent with the “self-medication hypothesis”, revisited from a neurobiological perspective, which asserts that the nicotine in tobacco may relieve or help manage specific psychiatric symptoms (5, 36).

Growing evidence suggests that the cognitive enhancement effects of nicotine may also contribute to the difficulty of quitting smoking, especially in individuals with psychiatric disorders who suffer from emotional, cognition and behavioral symptoms. They may also help explain why former smokers display increased risk of depression and substance use (37).

Numerous studies indicate the beneficial effect of nicotine on cognition in pathological conditions, also associated with cognitive deficit such as Alzheimer’s disease, Parkinson’s disease, schizophrenia, stress-induced anxiety, depression, and drug-induced memory impairment (38).

Such positive effects of nicotine and tobacco on neurocognition, mood, and other psychiatric symptoms constitute a mechanism that may contribute to the high coexistence of TUD and other mental disorders.

### Genes and environment

TUD has been genetically correlated with smoking and psychiatric traits (39) and environmental factors could predispose individuals to smoking and contribute to their susceptibility to develop an addiction. Indeed, a family history of tobacco consumption has been identified as a risk factor for TUD development. A non-negligible portion of this transgenerational risk can be presumed to be the result of developmental stage dependent epigenetic changes induced by nicotine (40). In addition, nicotine has been shown to trigger epigenetic changes in reward related (and other) brain areas that could form the basis of increased vulnerability to substance use (41) and other mental disorders (42). The growing importance of epigenetic modulation in mental health research notwithstanding, its translational potential is likely reserved to a distant future, while most of the research in this area has focused so far on the clear role of classic genetic polymorphisms.

Nicotine is the main psychoactive component of tobacco (43), and the nicotinic-cholinergic system has been implicated in different mental disorders (36). Therefore, finding that specific gene polymorphisms in key components of the nicotinic-cholinergic system, such as α5 nicotinic cholinergic receptor (CHRNA5) and the cytochrome P450 2A6 (CYP2A6), constitute measurable risk factors for TUD (44), is hardly surprising. Variants of CHRNA5 have been linked to compulsive smoking behavior, more difficulty in quitting, and relapse probability (36). Meanwhile, genotypic variations in CYP2A6, that influence the rate at which the enzyme metabolizes nicotine, have been associated with individual responses to NRT pharmacotherapy and thus TUD treatment success (44).

Based on a recent genome-wide multivariate association meta-analysis that breaks down the contribution of general and substance-specific loci, a polygenic addiction risk score was associated with SUD, other mental symptoms or psychiatric disorders, somatic conditions, and settings associated with addiction onset. Substance-specific loci (32 for tobacco) have been identified that include metabolic and receptor encoding genes. These findings provide insight into genetic risk sites for TUD that could be leveraged as treatment targets.

The potential impact of shared environmental factors on the emergence of dual disorders remains an important area in need of more research. For example, adverse childhood experiences (ACEs) increase not only the risk of SUD, including tobacco use but that of other mental disorders as well, including psychosis (45) and depression (46). A more granular description of overlapping triggers of dual disorders could help us improve DD prevention efforts. In depth, secondary analysis of ongoing longitudinal studies, beginning in childhood (ABCD study) (47) or at birth (HBCD study) (48) that collect a very dense array of environmental, genetic, neurobiological, social, psychological variables, as well as mental health outcomes could shed critical light in this context.

Finally, some endophenotypes, such as personality and behavioral traits, are related to addictive disorders. Some of these, are also observed in people with other mental disorders (34). Thus, both dual disorders could share some individual characteristics. These personality traits, along with the genes that regulate them, interact with the environment and substances such as tobacco, determining an individual vulnerability.

### Neurodevelopment

The adolescent brain is engaged in an active, experience-shaped process of maturation, which makes it particularly vulnerable to the effects of adverse events and toxic influences. Both preclinical and clinical studies show that exposure to nicotine during this period increases the risk of both cognitive deficits and psychiatric disorders later in life, partly due to alterations in acetylcholine and glutamate receptor signaling in the prefrontal cortex, one of the last brain areas to mature (49, 50). Some of the most compelling findings in this context include longitudinal studies showing early use of tobacco products to be associated with impaired cognitive performance and altered brain structure (51) as well as with higher rates of self-reported substance use and internalizing and externalizing problems (52). From the other direction, a recent study demonstrated that mental health problems predict the onset of tobacco use among youth and young adults, and across a wide range of specific tobacco products beyond cigarettes (8).

Developmental stage also interacts with genetic and environmental factors underlying the relationship between ADHD and the likelihood of smoking tobacco regularly and developing TUD (24), likely because nicotinic nAChR stimulation drives attention, which is necessary for learning and memory (53).

The growing neurophysiological evidence has the potential to help us better identify young individuals at risk of suffering TUD and/or other mental illnesses. For example, early-onset smokers display altered gray matter volumes in the vmPFC (54) or in the anterior insular complex (55), suggesting these changes could be predictive biomarkers of increased risk of tobacco use and addiction. While some research suggests adolescence is a risk factor for psychosis, the studies outlined above do not establish causality, but adolescents at ultra-high risk for psychotic disorders are likely to start smoking earlier and more intensely. The smoking prevalence in adolescents with ultra-high risk (UHR) for psychosis, was higher compared to healthy controls, with rates between 16.6 and 46% in UHR. In addition, UHR subjects were almost five times as likely to be heavy smokers compared to unaffected subjects (33, 56).

Since the arrival of vaping devices in 2007, young populations at risk of developing mental disorders face a new threat that increases their risk of TUD and other psychiatric disorders. A decades long declining trend in tobacco use has now been reversed partly thanks to exponential increases in the use of electronic cigarettes (e-cigarettes) (57) that cunningly cater to adolescents and young adults with enticing flavors and cool and easy to disguise devices (58). Since e-cigarettes are now the most used nicotine products among teenagers (59), researchers and clinicians should pay closer attention to vaping-related trends and the intricate associations between nicotine and mental disorders.

## Treatment

### Therapeutic options for people with TUD and other mental disorders

Current approved therapies for smoking cessation have modest long-term effects in achieving abstinence, even before considering patients suffering from DD. One of the main obstacles hampering most therapeutic approaches for TUD is considering it a behavioral problem, instead of a mental disorder (i.e., a brain disorder) likely linked to other mental disorders. This erroneous conceptualization gives rise to therapeutic proposals that are unmoored from the scientific evidence showing that tobacco, including one of its psychoactive molecules, nicotine, and other chemical derivates from tobacco, act on the brains of people with mental disorders in ways that differ from the ways in which they affect otherwise healthy people.

For people with DD, treatment must be bio-psycho-social, in that order. Biological interventions must consider the multifaceted roles of tobacco in the brain of people with mental disorders. This, combined with the lack of research and clinical guidelines on the treatment of TUD in patients with DD, underscores the need to prioritize investigations focused on this vulnerable population. In addition to the approved treatments, alternative harm reduction and/or replacement treatments, which operate through different mechanisms of action and offer various advantages, should be considered. All these modalities are described below.

### Pharmacological modulators on the endogenous nicotinic system

Nicotine is the main psychoactive component of tobacco. It binds to nicotinic acetylcholine receptors (nAChR) (involved in a broad range of physiological functions) (60), in several brain regions, where they modulate the release of various neurotransmitters, dopamine [DA] being the most studied. Dopamine’s activity in the mesolimbic dopaminergic system is the basis for the reward learning, motivational, and reinforcing effects of tobacco (61). Cessation of nicotine use can produce unpleasant withdrawal symptoms including irritability, insomnia, fatigue, headaches, and weight gain (62).

Available drugs with the ability to modulate the endogenous nicotinic system directly do so through their agonist, antagonist, or full/partial agonist effects. To date, four such drugs have been approved by the Food and Drugs Administration (FDA), or the European Medicine Agency (EMA) for TUD: nicotine, varenicline, cytisine, and bupropion. These therapies have shown efficacy but only short-term, with trials showing that nearly three quarters of the quitter’s relapse within a year (63). All these treatments have demonstrated a good safety and moderate efficacy profile for TUD in patients with other mental disorders (64).

*Nicotine replacement therapy (NRT)* aims to temporarily substitute nicotine from cigarettes to reduce both the motivation to smoke and withdrawal symptoms, thus facilitating the transition to reduced tobacco use or even abstinence (65). These therapies function as full agonists mimicking the effects of nicotine (62). Various forms of NRT are currently licensed, such as gum, transdermal patch, nasal spray, inhalator, or sublingual tablets, for smoking cessation in the general population (65). According to a systematic review, NRT has been demonstrated superior to placebo (odds ratio [OR]: 1.84; 95% confidence interval [95%CI]: 1.71 to 1.99) in smoking cessation (66), although there are very few studies in the population with mental disorders (67). Interestingly, NRT appears to be effective amongst individuals with fast, but not slow, CYP2A6-defined nicotine metabolism. Variations in treatment responses amongst individuals with dual TUD based on pharmacogenomic data may guide future personalized treatment (44). Some of the NRT advantages are high adherence and the possibility to combine it with other therapies for TUD (4).

*Bupropion* is a drug that partially inhibits DA and norepinephrine reuptake in the brain reward centers and acts as an antagonist of nAChR activity, decreasing the rewarding properties of nicotine (68). However, despite some studies indicating that bupropion can alleviate some withdrawal symptoms, these effects appear modest (62, 68). Nonetheless, bupropion could be used in combination with NRT. Interestingly, and in contrast to NRT, the positive effects of bupropion on the likelihood of relapse don’t appear to depend on CYP2A6 genotype (i.e., nicotine metabolism rates) (44).

*Varenicline* is a partial agonist of α4β2 nicotinic receptors that has shown to be safe and effective in maintaining abstinence in smokers (62). As a partial agonist, in the presence of a full agonist like nicotine, varenicline will act as an antagonist by competing in nAChR binding, thus attenuating the effects of nicotine in the reward circuit. But, in the absence of nicotine, its partial agonism could also attenuate withdrawal symptoms (62). Therefore, varenicline has the potential to block nicotine reward, minimize craving, and prevent withdrawal symptoms. Indeed, varenicline appears to be significantly more effective than placebo in assisting people with severe mental dual disorders to reduce or quit smoking (69). In July 2021, production of varenicline tartrate was stopped due to elevated nitrosamine levels and it is possible it could be replaced by the advent of generics such as Apo-varenicline.

Studies have reported a superior efficacy of both bupropion and varenicline relative to placebo in smoking cessation (OR: 1.82; 95% CI: 1.60 to 2.06 and OR: 2.88; 95% CI: 2.40 to 3.47) (66). Head-to-head comparisons have shown that bupropion and NRT are equally effective (OR: 0.99; 95% CI: 0.86 to 1.13), whereas varenicline appears to be superior than NRT (OR: 1.57; 95% CI: 1.29 to 1.91) and bupropion (OR: 1.59; 95% CI: 1.29 to 1.96) (66).

*Cytisine* is also a partial agonist of the α4β2 nAChR responsible for central effects of nicotine that aims to reduce smoking rates and withdrawal symptoms. Oral cytisine reaches peak concentration two hours post dose and is excreted unchanged renally without hepatic metabolism, lowering the risk of drug interactions. Oral cytisine has a shorter half-life (4.8 v. 17 h) and treatment course (3.5 v. 12 wk.) than varenicline (70). Although not licensed in the US, cytisine is used in some European countries to aid smoking cessation, but its traditional dosing regimen and treatment duration may not be optimal (71). However, the use of cytisine for the treatment of tobacco use in western countries is very recent and there are no studies in dual TUD subpopulations. However, there is reason to expect that cytisine could yield similar results to those obtained with varenicline (4), further expanding the pharmacological toolkit.

### Results from clinical trials

Regarding the clinical efficacy of these pharmacotherapies, the EAGLES trial (Evaluating Adverse Events in a Global Smoking Cessation Study) is the largest smoking cessation study performed in adults (72, 73). It included 8,144 smokers with and without mental disorders, and compared the safety and efficacy profiles of varenicline, NRT (nicotine patch), bupropion, and placebo (72). This study did not find a significant increase in neuropsychiatric adverse events, such as anxiety, depression, agitation, or panic, in adults treated with varenicline or bupropion compared to those treated with NRT and placebo, in both psychiatric and non-psychiatric subpopulations. Varenicline showed higher efficacy with greater abstinence rates compared to bupropion or NRT (72). Bupropion and NRT had similar efficacy in achieving abstinence, which was superior relative to placebo. A secondary analysis of safety and efficacy outcomes, focusing on TUD with other mental disorders was performed (73). Patients were divided into three groups according to their other mental disorder (psychotic, anxiety, and mood disorders). Varenicline, bupropion and NRT were well-tolerated and effective in persons with TUD and other mental disorders, without differences among the three defined groups (73). Bupropion and NRT had similar efficacy, superior to placebo, but lower than varenicline. A meta-analysis assessing the efficacy and tolerability of pharmacotherapy for TUD in adults with serious mental disorders, revealed that both bupropion and varenicline were more effective than placebo (OR: 4.51; 95% CI. 1.45 to 14.04 and OR: 5.17; 95% CI: 1.78 to 15.06, respectively) (74).

Moreover, three-month maintenance therapy with varenicline appears to maintain tobacco abstinence rates in patients with schizophrenia and bipolar disorder (75). In patients with TUD and schizophrenia, varenicline seems to be efficient in reducing tobacco use without worsening the psychiatric symptoms (76). Along with these effects on reducing tobacco use, varenicline has been shown to improve some psychiatric symptoms, such as hyperactivity in an ADHD subclinical population, reduce withdrawal-related depression in smokers with depressive symptoms, and reduce anxiety (77–79).

### Non-nicotine-based treatment strategies

*Repetitive transcranial magnetic stimulation* (rTMS) is a non-invasive, drug-free, neural-circuit-based therapeutic tool that was recently cleared by the United States Food and Drug Associate for the treatment of tobacco smoking (80). Initial results have shown that rTMS could be useful in addiction treatment. In fact, the first large multicenter study of brain stimulation in addiction, which led to the first FDA authorization for rTMS as an aid in smoking cessation, pointed out that the efficacy of rTMS resulted in a reduction in cigarette consumption and craving in TUD patients (80). The anterior insular cortex has been identified by preclinical and clinical studies as a critical target for addiction treatment and is a key region mediating compulsive drug-taking (including tobacco) (55). The functions of the insula can be modulated non-invasively by deep brain TMS stimulation, although there are no studies in populations with severe mental disorders (81). Although this is a step forward in the search for new TUD treatments, it is still limited to short-term efficacy and there are no data on its use in people with dual TUD (82).

*Psilocybin*, a serotonin 5HT2 receptor agonist and possible glutamatergic action, is being investigated to treat several DD. In a recent open-label pilot study (N = 15) patients with TUD were administered moderate to high doses (20 and 30 mg/70 kg) of psilocybin, in combination with cognitive behavioral therapy (CBT). The combination of psilocybin and CBT resulted in significantly higher smoking abstinence rates a year later (83).

Ketamine and esketamine are also psychoactive substances recently investigated for various DD. In animal studies, ketamine significantly reduced nicotine self-administration in a dose-dependent manner. In addition, a differential sensitivity between sexes was observed, since male rats responded to a lower dose of ketamine and with a larger effect size than female rats. It is concluded that the modulation of the glutamatergic receptor may offer a novel and potentially gender-dependent intervention in TUD (84).

*Monoamine oxidases (MAO)* are enzymes that metabolize DA, influencing their activity in dopaminergic reward brain circuits. This enzyme has been related to TUD, as its activity was found ~30% reduced in brains and bodies of long-term smokers. As we mentioned earlier, some chemicals in tobacco have an MAO-inhibiting effect, which could explain its antidepressant and other mood effects. For this reason, some MAO inhibitors have been tried for TUD (85).

### Alternative treatment strategies for dual TUD

Although most patients with TUD say they want to quit smoking, less than 5% of them can do so without help (86). To achieve successful treatment, it is necessary to first recognize that TUD is a mental disorder, and therefore a brain disorder, related to other mental disorders. Treatment should set realistic goals for these dual TUD patients, such as reducing tobacco use on the way to cessation. For those patients with dual TUD who are unable to quit but must reduce their tobacco use due to health concerns, alternative smoking products should be considered (86).

In this context, it is relevant to consider the emergence of electronic cigarettes (ECs), a novel array of nicotine delivery products, “pod” based devices, that use nicotine liquids (87). According to searches of the Cochrane Tobacco Addiction Group Specialized Register to examine the safety, tolerability, and effectiveness of using electronic cigarettes (ECs) to help people who smoke tobacco achieve long-term abstinence from tobacco, there is high-certainty evidence that ECs with nicotine increase quit rates compared with NRT and moderate-certainty evidence that they increase quit rates compared with ECs without nicotine, although this search does not distinguish people with other mental disorders (88, 89).

On the other hand, it is important to recognize and understand EC’s addictive potential and safety profile to regulate their use and evaluate their potential as an aid for smoking cessation. At the same time, opinions in the scientific community regarding the potential use of ECs for TUD, are divided, although this controversy does not usually consider people with dual TUD. This point is particularly important, considering nicotine’s potential therapeutic aspects for at least some dual TUD patients, the notion of tobacco cessation as the only acceptable endpoint in these cases is an important research question moving forward. In fact, some studies have suggested that replacing traditional tobacco with ECs could increase life expectancy (90, 91). Therefore, even in the most pessimistic scenario, substituting traditional cigarettes with ECs might lead to substantial health benefits.

A smoking cessation study in the UK found that the abstinence rate at the one-year mark was almost double in the ECs vs the NRT group (18.0% vs. 9.9%, respectively), and that the former also had higher adherence (92). The most frequently reported adverse reactions were throat/mouth irritation in the e-cigarette group, with little difference in the reporting of severe nausea or severe throat/mouth irritation between the two groups (92).

A recent randomized clinical trial found that varenicline and nicotine-containing ECs were both effective in helping individuals in quitting smoking conventional cigarettes for up to 6 months (93).

In another recent randomized controlled trial, biomarkers of exposure to harmful components and biomarkers of potential harm were compared in adult smokers who switched to e-cigarettes versus smokers who continued smoking for 24 weeks. The results strongly suggest significant reductions in biomarkers of exposure (excluding nicotine) accompanied by favorable changes in several biomarkers of potential harm (oxidative stress and inflammation), including lung function. The totality of the evidence suggests that exclusive e-cigarette use may pose lower health risks compared with conventional smoking (94).

Regarding risks for second-hand exposure, when looking at the safety of children exposed to second-hand tobacco or e-cigarettes, recent findings suggest that switching from smoking to vaping indoors can substantially reduce, but not eliminate, children’s second-hand exposure to nicotine and other harmful substances (95).

Only a few studies have analyzed the use of ECs in dual TUD subpopulations. A small uncontrolled study was conducted to investigate the efficacy of ECs for smoking reduction in subjects with schizophrenia (96). Half of the patients who were using e-cigarettes for 12 weeks reduced their smoking by 50%, and 14% quit completely without exacerbating psychiatric symptoms. More recently, a 24-week pilot study investigated the use of ECs to reduce smoking in subjects with psychotic disorder (97). Despite some limitations, the study supported the acceptability of ECs without a negative impact on mental health.

Therefore, although the long-term health effects of these new nicotine delivery products are still unknown, their consumption might reduce exposure to the harmful components of traditional cigarettes. These alternatives merit rigorous investigations as we consider the best treatments for dual TUD patients.

Prior to 2016, when FDA extended the law for New Tobacco Products to ECs, those were not regulated. However, since then, any e-cigarette product on the market must apply to the FDA for permission and submit detailed information about its ingredients and manufacturing process. Also, such products will be subject to FDA inspections. In October 2021, FDA authorized the marketing of three new tobacco products, being the first set of electronic nicotine delivery system products ever authorized by FDA. In Europe, similar measures have not been promoted yet, although efforts are being made to provide users guarantees. In the UK, the Medicines and Healthcare Products Regulatory Agency (MHRA) has updated guidance, allowing medically licensed vaping products to be prescribed to smokers who want to quit, and e-cigarette manufacturers can submit their products for the same regulatory approvals process as drug companies for use on the NHS in England.

Some researchers have proposed smoking treatment trials utilizing ECs in people with other mental disorders. To this end, the UK has already begun allowing companies to submit their products for approval as medically licensed ECs that can be prescribed as smoking cessation or harm reduction aids. The proposal is timely, backed by evidence, and aims to save hundreds of thousands of lives (98), although the long-term effects of ECs use are still unknown.

Another alternative nicotine delivery product is “snus” (Swedish-type moist snuff), which is smokeless tobacco whose more common application method is by portion-packed tobacco in small sachets (99). The sale of snus was prohibited in the EU, except for Sweden, Norway, and Switzerland, which have special derogations. The consumption in these regions has provided epidemiological data supporting its long-term safety. According to available evidence from these countries, there is little support for the existence of any major adverse health effect of snus, which appears to be significantly safer than smoking combusted tobacco (99). Moreover, there is no evidence of its consumption encouraging smoking initiation or discouraging cessation. There is also no reliable evidence that snus consumption can affect the onset of psychiatric disorders (99).

*Heated Tobacco without combustion (HT)*: despite all the tools for the treatment of TUD, there is still a significant population with severe mental disorders who are unable to achieve abstinence or reduce tobacco use. At this point it is important to consider that other chemicals in tobacco, beyond nicotine, have an impact on the brain of people suffering other mental disorders. This observation may help explain the limited efficacy of cholinergic/nicotinic system modulators in some groups of patients. Tobacco exerts some of its effects through non-nicotinic means, for example, the MAO-inhibiting effect derived from other chemicals in tobacco could act as an antidepressant (85).

The availability of new products that do not replace only nicotine but replace conventional tobacco could be of particular interest as we develop new therapeutic tools to treat dual TUD in populations with severe mental disorders. Heated cigarettes are novel nicotine and tobacco delivery systems that use an electronic heating method to warm tobacco at a low temperature without combustion (4, 100). Traditional cigarette smoke emissions are mainly generated by the distillation, pyrolysis, and combustion reactions that occur when tobacco is burned. However, studies have shown that heating tobacco to temperatures below the pyrolysis and combustion thresholds has the potential to decrease some of the toxic substances found in combusted tobacco, which is heated over 600°C.

Some studies have evaluated the potential benefits of using heated tobacco devices showing that they could reduce exposure to harmful toxins derived from tobacco combustion, as well as reduce the levels of some risk markers (i.e., carboxyhemoglobin and mercapturic acids) (101–104). This has also been observed in a recent systematic review, which suggested that heated tobacco presents a reduced risk of chronic diseases, including respiratory and cardiovascular diseases and cancer compared to traditional cigarettes (105). Similarly, in a recent narrative review, authors analyzed a total of 52 studies that provided evidence that heated tobacco could reduce the emission of harmful and potentially harmful constituents and the exposure to toxic substances (106). Therefore, heated tobacco may have a lower biological and clinical impact compared to traditional cigarettes.

Recently the first randomized controlled trial comparing quit rates between HT and ECs among people who smoke and do not intend to quit was published. Switching to HT elicited a marked reduction in cigarette consumption among people who smoke and do not intend to quit, which was comparable to refillable ECs. User experience and risk perception were similar between HT and ECs under investigation (100).

These novel devices might be useful in TUD patients to reduce the use of conventional combusted tobacco and, therefore, its potentially negative health consequences. In patients with dual TUD, studies have suggested that heated tobacco can reduce the consumption of daily cigarettes with a good adherence (107). Indeed, in recent years, these new nicotine delivery products have been commercialized as less harmful alternatives to traditional combusted tobacco. However, better studies are needed to ensure that the introduction of these new devices does not increase tobacco consumption. For example, the sample sizes of the published studies are small, and further evaluation in larger populations is required to establish the evidence for potential approval, oversight, and regulation by regulatory agencies such as FDA and EMA (86).

## Clinical implications

We are witnessing the emergence of precision medicine in psychiatry. This perspective hinges on the fact that psychoactive substances have different effects on different brains, underpinning individual differences in mental health and illness (34). Recognizing the needs of specific populations is a public health imperative.

People suffering from TUD and other mental disorders present a particularly difficult challenge as they represent a highly heterogeneous population with clear clinical subcategories and expectedly different treatment trajectories. For example, in some studies, smoking cessation has been associated with lower depression, anxiety, and stress, and with an improved quality of life and emotional state (108, 109). However, these clinical findings do not explain why most people with serious mental disorders find it so difficult to remain abstinent from tobacco, perhaps those studies are related to phenotypes of less severe mental disorders (6, 110). On the other hand, the self-medication hypothesis leads to concerns that aggressively treating smoking may worsen the symptoms of other mental disorders (including other SUD) (6, 111). If this were true, it could mean an important limitation for TUD treatment in patients with other mental disorders, while opening an important discussion in the DD field. Therefore, since the treatment of TUD could theoretically lead to both improvement and worsening of DD, an integrative, rational, and personalized approach will be necessary to understand the role of tobacco in mental disorders.

The term “cessation” may exclude alternative treatment options beneficial to people with severe mental disorders, such as harm reduction or the less stigmatizing term “substitution therapy” (3).

Some people have proposed getting rid of commercial tobacco products in this century and hastening the demise of the tobacco industry and some tobacco companies are responding with a strategy of switching to less harmful nicotine delivery systems (112).

Given the role of the cholinergic/nicotinic system in mental functions and, therefore, in mental disorders, it is worth asking whether all persons with severe mental disorders could live without nicotine.

## Practical proposals in the real world

In the real world, the treatment of dual disorders should be personalized and propose realistic goals, removed from moral concepts such as total cessation of tobacco and its derivatives as the only option for all people with dual TUD.

TUD should be treated from the beginning with other mental disorders. Bio-psycho-social treatment should be proposed in that order. Regarding TUD, combined treatment with medications approved by regulatory agencies (nicotine, bupropion, cytisine, varenicline or TMS) and others that release nicotine (substitution treatment) should be personalized and proposed at different stages of the therapeutic process.

## Conclusions

According to epidemiological studies, TUD is expected rather than an exceptional feature among people with mental disorders. The probability of having TUD is higher in patients with other severe mental disorders. Yet, despite a growing interest among the scientific community in the management of dual TUD in patients with severe mental disorders, this subpopulation still has higher smoking prevalence and lower quit rates (6).

The high symptomatic prevalence of TUD and other mental disorders strongly suggests that Dual TUD is not due solely to random or coincidental factors. It seems reasonable to hypothesize that both clinical conditions share common genetic, neurobiological, developmental, and/or environmental bases and are causally linked. Unfortunately, treatment is often directed at the TUD as a behavioral problem, whereas its highly heterogeneous nature warrants personalized approaches.

Dual TUD patients stand to benefit from precision psychiatry strategies that leverage some combination of predictive polygenic scores (34), pharmacogenomic tools, environmental and psychosocial interventions, and harm reduction approaches. Importantly, the knowledge of clinicians with experience in the concept of DD should be disseminated to improve program outcomes. It is high time for increasing specialized training in TUD and DD.
